# Early-Onset Hyponatremia Presenting as Syndrome of Inappropriate Antidiuretic Hormone Secretion (SIADH) Post-transsphenoidal Pituitary Resection

**DOI:** 10.7759/cureus.105276

**Published:** 2026-03-15

**Authors:** Eileen M Leach, Ashley Tuin, Allison Christy, Maddison Weber, Khalid Bashir

**Affiliations:** 1 Department of Internal Medicine, Creighton University School of Medicine, Omaha, USA; 2 Department of Nephrology, Creighton University School of Medicine, Omaha, USA

**Keywords:** antidiuretic hormone, nonfunctioning pituitary neuroendocrine tumor, postoperative complication, symptomatic hyponatremia, syndrome of inappropriate antidiuretic hormone secretion (siadh), transsphenoidal resection

## Abstract

Syndrome of inappropriate antidiuretic hormone secretion (SIADH) is a disorder in which an excessive amount of antidiuretic hormone (ADH) is released, leading to water retention and subsequent hyponatremia. Delayed hyponatremia, typically beginning on postoperative day (POD) 4, is a well-recognized complication following transsphenoidal resection of pituitary tumors and is thought to result from excess release of stored ADH due to surgical trauma. In this rare case of early symptomatic hyponatremia secondary to SIADH, a 61-year-old postmenopausal woman with no significant past medical history developed symptomatic hyponatremia on POD 1 after elective transsphenoidal resection of a nonfunctioning pituitary neuroendocrine tumor. Her serum sodium reached a nadir of 126 mmol/L on POD 2. Urine osmolality was 961 mOsm/kg on POD 1, consistent with SIADH. She was treated with fluid restriction, intravenous 3% hypertonic saline, and salt tablets, with improvement in serum sodium prior to discharge on POD 4 on fluid restriction and salt tablets. This case highlights the importance of early clinical vigilance for hyponatremia following pituitary resection, as symptomatic hyponatremia can rarely occur in the early postoperative period.

## Introduction

Hyponatremia is a well-recognized complication following transsphenoidal resection of pituitary tumors. It typically occurs secondary to syndrome of inappropriate antidiuretic hormone secretion (SIADH) [1]. Normally, antidiuretic hormone (ADH) helps maintain fluid and electrolyte homeostasis by regulating water reabsorption in the kidneys. SIADH occurs when an excessive amount of ADH is released, leading to water retention. The relative excess of water results in hyponatremia [2].

In addition to SIADH, other disorders of sodium homeostasis, including arginine vasopressin deficiency (AVP-D) and cerebral salt-wasting syndrome (CSWS), have been observed after transsphenoidal pituitary resection. AVP-D, also known as diabetes insipidus, typically occurs earlier than SIADH [1]. In this condition, patients cannot produce or secrete ADH, leading to polyuria and either eunatremia or hypernatremia. CSWS is a rarer, less understood cause of hyponatremia that occurs due to a central nervous system insult; unlike SIADH, it leads to hypovolemia [1].

The most common onset of SIADH is approximately one week after surgery, but it has rarely been reported on postoperative day (POD) 1 [1-5]. Larger studies suggest postoperative hyponatremia occurs in 7-15% of patients, with one study reporting an incidence of 24% [2,3,6]. Risk factors for postoperative hyponatremia are not well defined but may include female gender, older age, and preoperative hyponatremia or hypopituitarism [1,2]. The pathophysiology of postoperative hyponatremia is most commonly attributed to injured nerve terminals causing excess release of stored ADH from the posterior pituitary [1].

This case describes a rare early presentation of symptomatic hyponatremia secondary to SIADH following transsphenoidal resection of an anterior nonfunctioning pituitary neuroendocrine tumor (NF-PitNET), highlighting the need for clinical vigilance and early sodium monitoring in the postoperative period.

## Case presentation

**Figure 1:**
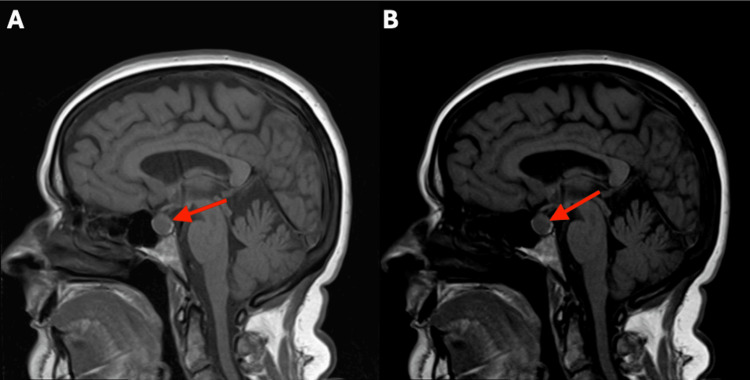
Sagittal T1-weighted (A) and T2-weighted (B) MRIs showing NF-PitNET at the time of presentation The red arrow indicates the NF-PitNET, measuring 1.1 cm × 1.3 cm. NF-PitNET, nonfunctioning pituitary neuroendocrine tumor

A 61-year-old postmenopausal woman with a past medical history of migraine headaches underwent elective transsphenoidal resection of an NF-PitNET in July 2025. The pituitary lesion was initially identified on MRI in April 2021 during a workup for headaches, at which time it measured 1.1 cm × 1.3 cm (Figure 1). Endocrine workup at that time revealed normal levels of adrenocorticotropic hormone, random cortisol, prolactin, follicle-stimulating hormone (FSH), luteinizing hormone, insulin-like growth factor-1, thyroid-stimulating hormone (TSH), and free thyroxine (T4).

**Figure 2:**
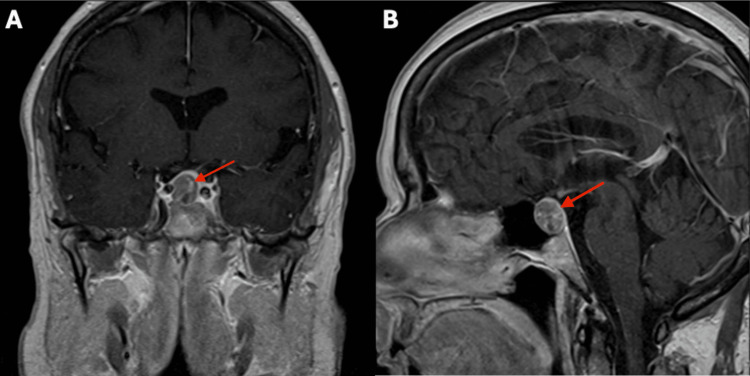
Coronal (A) and sagittal (B) T1-weighted MRIs showing NF-PitNET prior to resection The red arrow indicates the NF-PitNET, measuring 1.2 cm × 1.5 cm. NF-PitNET, nonfunctioning pituitary neuroendocrine tumor

**Figure 3:**
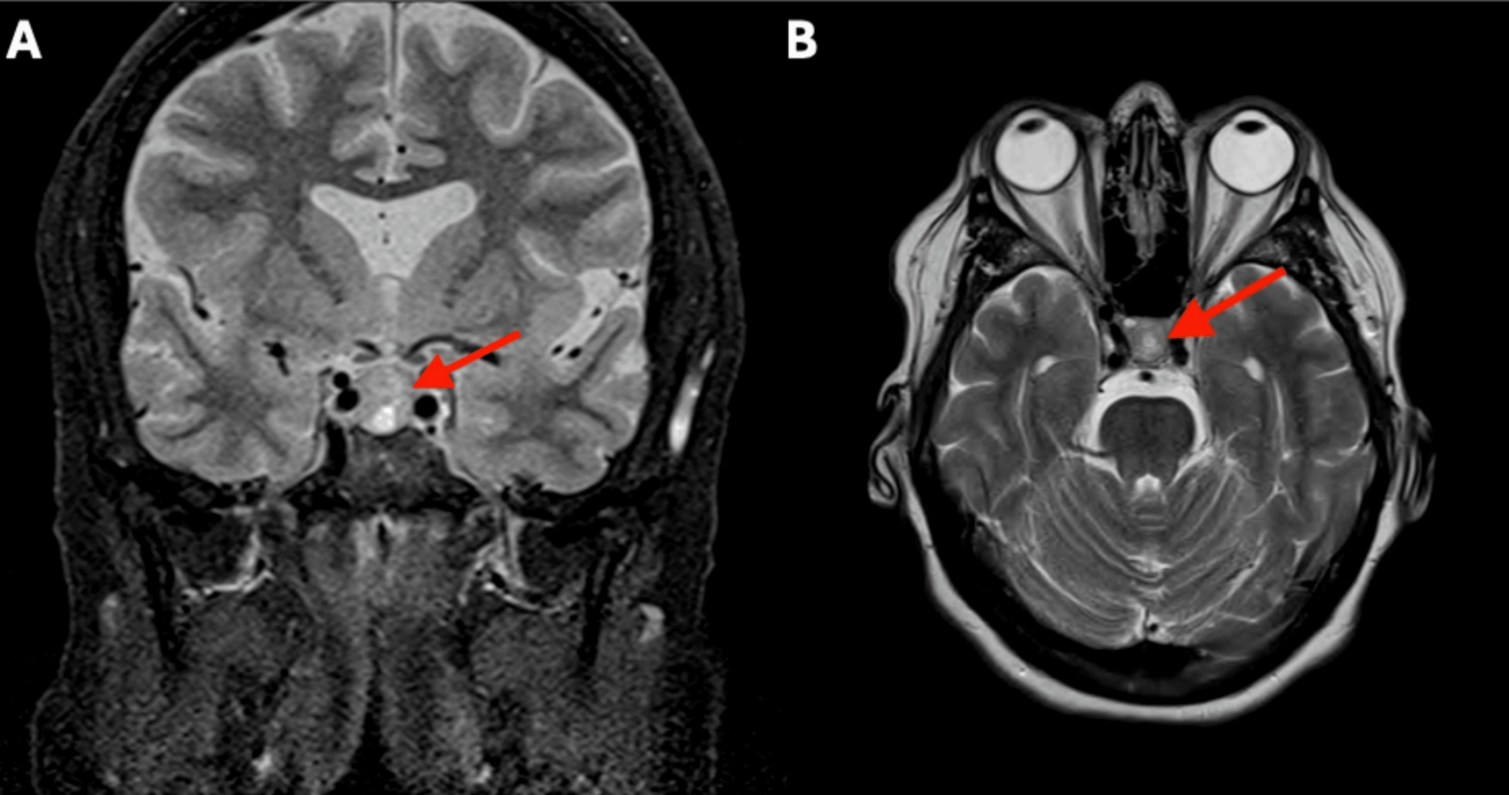
Coronal (A) and axial (B) T2-weighted MRIs showing NF-PitNET prior to resection The red arrow indicates the NF-PitNET, measuring 1.2 cm × 1.5 cm. NF-PitNET, nonfunctioning pituitary neuroendocrine tumor

Serial MRI imaging demonstrated gradual enlargement of the NF-PitNET over four years. The patient and her provider opted for surgical removal rather than continued serial imaging, at which point the NF-PitNET measured 1.5 cm × 1.2 cm × 1.2 cm (Figure 2, Figure 3). 

The patient underwent endoscopic transsphenoidal resection of her pituitary mass. The surgeons accessed the sella via the sphenoid sinus and removed the pituitary mass with the aid of a ring curette. At the end of the procedure, a small cerebrospinal fluid leak from the diaphragma sellae was noted and managed using a collagen matrix (DuraGen) and surgical sealant. No other acute surgical complications were reported.

**Figure 4:**
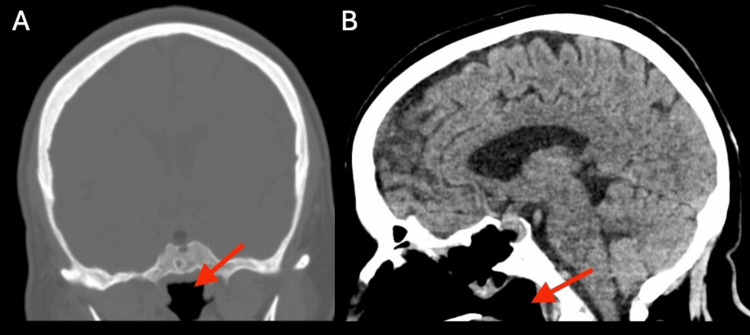
Coronal (A) and sagittal (B) non-contrast head CT performed 10 hours after transsphenoidal resection of the pituitary neuroendocrine tumor The red arrow indicates residual mass versus intrasellar hemorrhage measuring 2.2 cm × 1.3 cm × 1.6 cm.

The initial postoperative CT scan demonstrated post-surgical changes with residual mass versus intrasellar hemorrhage, measuring 2.2 cm × 1.3 cm × 1.6 cm (Figure 4). The resected tissue was sent for pathological examination. Immunohistochemical staining showed diffuse positivity for steroidogenic factor 1 and focal positivity for FSH, consistent with gonadotroph differentiation. Immediate postoperative laboratory values were within normal limits, including a serum sodium of 136 mmol/L (Table 1).

**Table 1:** Serum sodium, serum osmolality, and urine osmolality trends by POD in a euvolemic patient Serum sodium was measured every six hours, while a basic metabolic panel was obtained each morning. Serum osmolality was calculated using the basic metabolic panel results rather than the lowest serum sodium, which was not necessarily drawn from the morning panel. POD, postoperative day

POD	Lowest serum sodium (mmol/L)	Reference range (mmol/L)	Serum osmolality on morning lab draw (mOsm/kg)	Reference range (mOsm/kg)	Urine osmolality (mOsm/kg)	Reference range (mOsm/kg)
POD 0	136	135-145	285	285-295	634	300-1000
POD 1	133	135-145	284	285-295	961	300-1000
POD 2	126	135-145	270	285-295	533	300-1000
POD 3	128	135-145	266	285-295	303	300-1000
POD 4	132	135-145	273	285-295	380	300-1000

The patient developed symptoms of headache, nausea, and generalized malaise over the next 48 hours, with a nadir serum sodium of 126 mmol/L on the evening of POD 2. Urine osmolality peaked on POD 1 at 961 mOsm/kg (Table 1). On POD 1, morning cortisol was 15.9 µg/dL (reference range 5.3-22.4 µg/dL), excluding adrenal insufficiency. Laboratory values on POD 2 revealed urine sodium of 119 mmol/L, urine osmolality of 533 mOsm/kg, TSH of 0.624 µIU/mL, free T4 of 1.51 ng/dL, and triiodothyronine of 2.9 pg/mL (Table 2).

**Table 2:** POD 2 laboratory values in a euvolemic patient POD, postoperative day; T3, triiodothyronine; T4, thyroxine; TSH, thyroid-stimulating hormone

Test	Value	Reference range
Serum		
Sodium	126 mmol/L	135-145 mmol/L
TSH	0.624 µIU/mL	0.550-4.780 µIU/mL
Free T4	1.51 ng/dL	0.70-1.45 ng/dL
Free T3	2.9 pg/mL	2.3-4.2 pg/mL
Urine		
Sodium	119 mmol/L	20-110 mmol/L
Osmolality	533 mOsm/kg	300-1000 mOsm/kg

On physical examination, the patient appeared euvolemic, with no clear evidence of fluid overload. Fluid status is a crucial differentiator between SIADH and CSWS: patients with SIADH are euvolemic, whereas those with CSWS are hypovolemic and have a net negative fluid balance [7]. The patient’s intake and output were monitored by nursing. Charting suggested a net output on POD 2-4; however, she gained 5.4 kg over the course of her admission. Given that the more objective measure (weight) indicated a positive fluid balance, it is likely that intake and output were charted inaccurately, making CSWS less likely than SIADH (Table 3).

**Table 3:** Trends in patient weight and intake/output by POD PO, per os (by mouth); POD, postoperative day

POD	Weight (kg)	Intake (cc)	Output (cc)	Net intake/output (cc)
POD 0	85.1	Strict intake and output not recorded		
POD 1	89			
POD 2	Not recorded	400 PO, 35 IV	2050 urine	-1615
POD 3	90.8	1100 PO, 92 IV	3600 urine, 2 stools	-2408
POD 4	90.5	500 PO	620 urine	-120

Another differentiator between SIADH and CSWS is that the fractional excretion of urate (FEurate) is initially elevated in both conditions but remains elevated after correction of hyponatremia in patients with CSWS. FEurate was not measured in this patient. She was not taking any medications known to cause hyponatremia prior to, during, or after surgery. With other causes ruled out, she met the Bartter-Schwartz criteria for SIADH (Table 4) [8]. 

**Table 4:** Comparison of the patient’s presentation on POD 2 to the Bartter-Schwartz criteria for SIADH The Bartter-Schwartz criteria have historically been used to diagnose SIADH [8]. POD, postoperative day; SIADH, syndrome of inappropriate antidiuretic hormone secretion

Patient values on POD 2 when symptoms began	Bartter and Schwartz criteria for SIADH
Serum osmolality = 270 mOsm/kg	Serum osmolality <275 mOsm/kg
Urine osmolality = 533 mOsm/kg	Urine osmolality >100 mOsm/kg
Urine sodium = 119 mmol/L	Urine sodium >30 mmol/L
Appears euvolemic on clinical exam	Euvolemia
No evidence of diuretic use, hypothyroidism, adrenal insufficiency, hyperprolactinemia, hyperlipidemia, or hyperglycemia	No other causes of hyponatremia

As the presentation was consistent with postoperative SIADH, the patient was managed with fluid restriction of 1.5 L per day and three doses of 100 mL of 3% hypertonic saline administered at a rate of 25 mL/h. Six hours after the first dose of 3% hypertonic saline, serum sodium was 126 mmol/L. By 24 hours, serum sodium had risen to 131 mmol/L. Serum sodium continued to improve on POD 4 (132 mmol/L) with a urine osmolality of 380 mOsm/L. The improvement in hyponatremia with fluid restriction is more consistent with SIADH than with CSWS, as CSWS typically worsens with fluid restriction.

**Figure 5:**
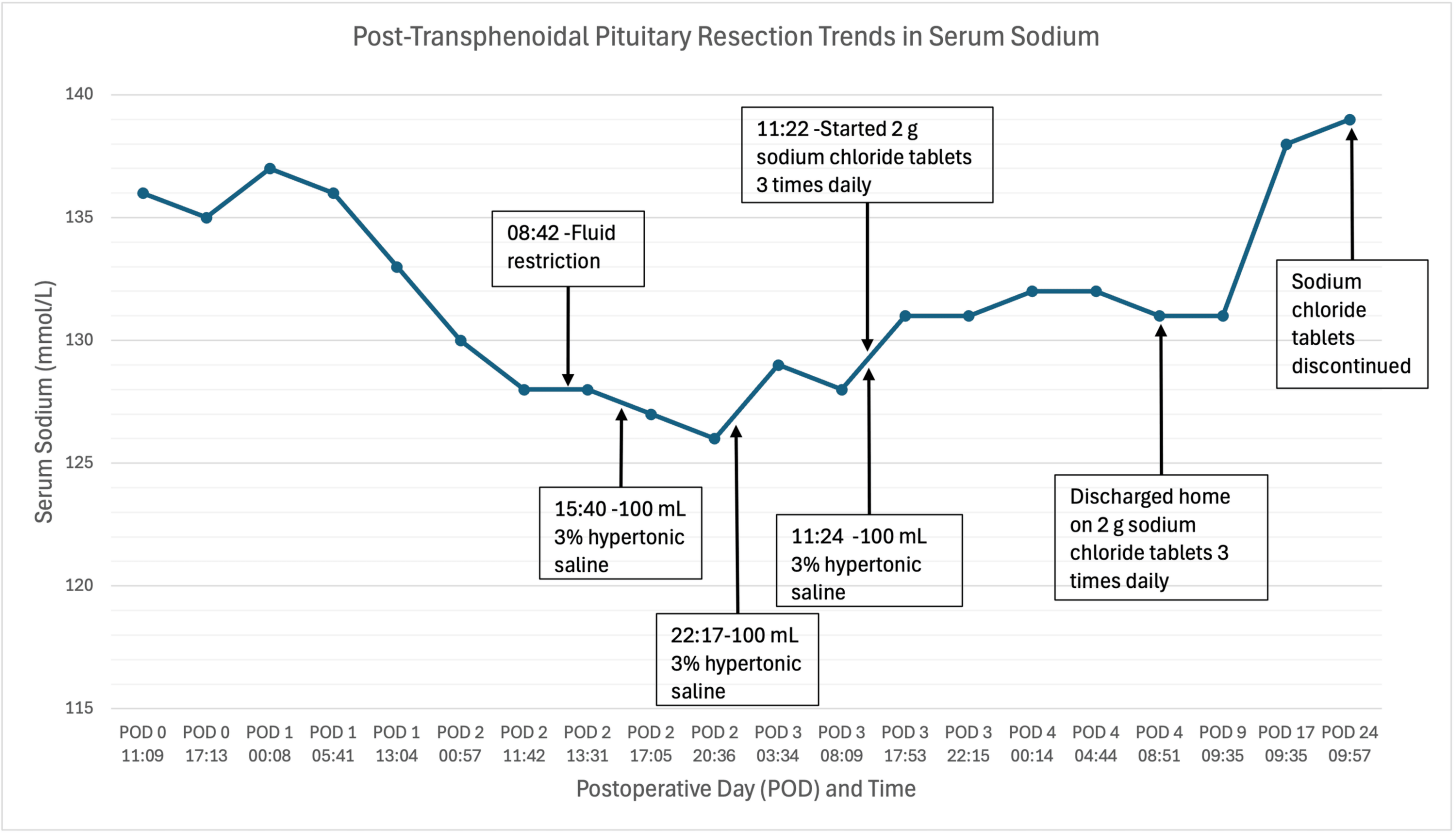
Sodium levels and interventions in the postoperative period following transsphenoidal resection of an NF-PitNET NF-PitNET, nonfunctioning pituitary neuroendocrine tumor; POD, postoperative day

The patient was transitioned from hypertonic saline to oral salt tablets on POD 3. On POD 4, she was discharged in stable condition on 2 g salt tablets three times per day with a fluid restriction of 1.5 L per day, with plans for outpatient follow-up. After discharge, her serum sodium remained above 131 mmol/L, and the sodium tablets were slowly tapered by POD 24 (Figure 5).

**Figure 6:**
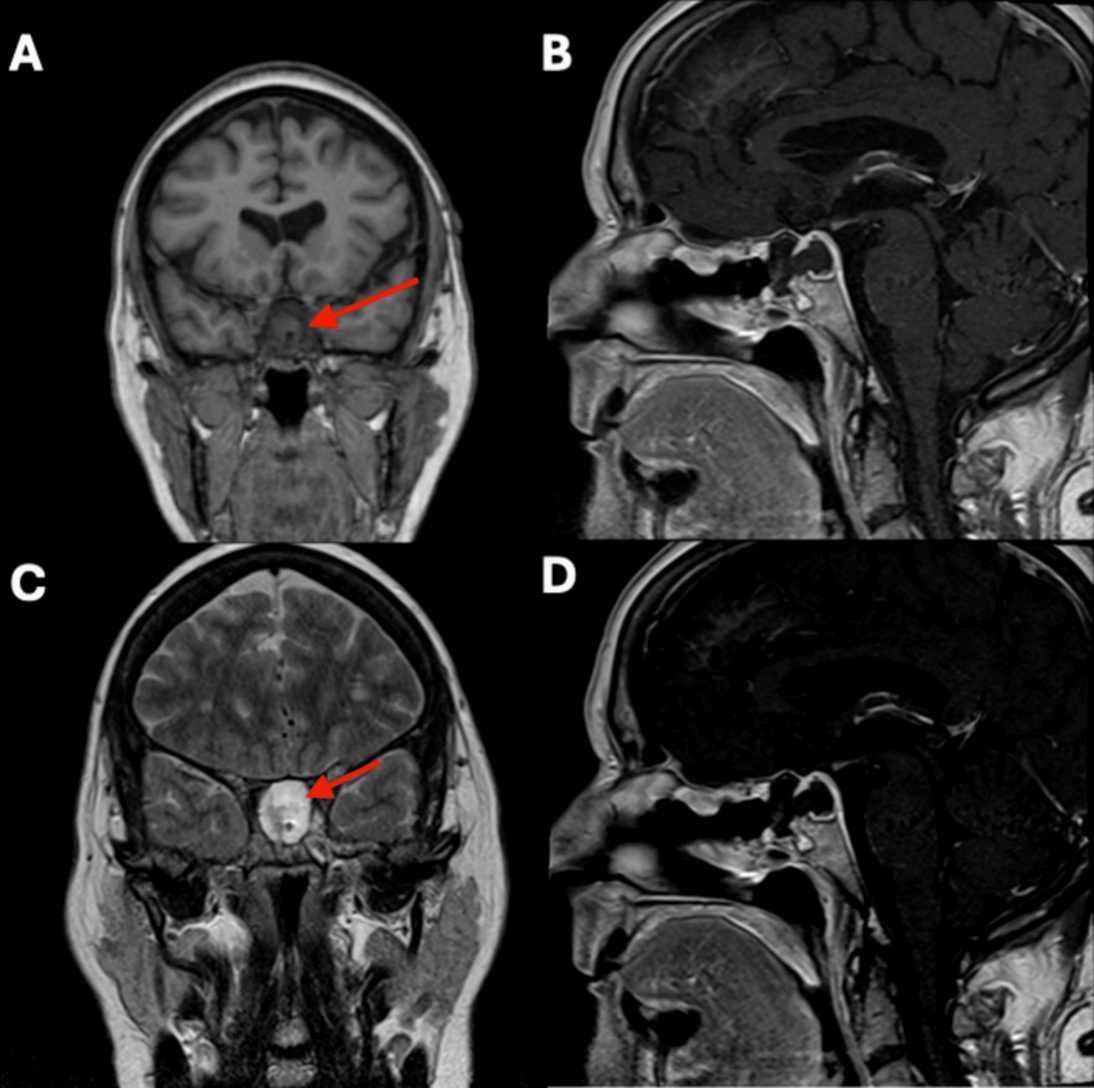
Coronal (A) and sagittal (B) T1-weighted MRIs and coronal (C) and sagittal (D) T2-weighted MRIs one month post-transsphenoidal resection of the pituitary neuroendocrine tumor The red arrow indicates the fluid-filled sella turcica and suprasellar cistern, measuring 1.8 cm cephalocaudally, without a solid mass.

One month postoperatively, MRI revealed expected postoperative changes, including a fluid-filled sella turcica and suprasellar cistern but no solid mass (Figure 6). A follow-up MRI performed five months postoperatively showed that the hypoenhancing area in the sella turcica could not be definitively differentiated as residual tumor or post-surgical cavity, but its size had decreased from 1.8 cm to 0.7 cm cephalocaudally compared to the one-month MRI. At her five-month follow-up, the patient’s serum sodium was 140 mmol/L without medication. Neurosurgery plans to repeat imaging one year postoperatively to monitor the area of hypoenhancement. 

## Discussion

Hyponatremia is a common complication of transsphenoidal resection of pituitary tumors. While the incidence and mechanism are not fully established, postoperative hyponatremia typically develops after at least three days, most commonly on day 7 [1,3,6]. ADH is produced in the hypothalamus and transported along the hypothalamic-hypophyseal tract for storage in the posterior pituitary. ADH promotes water reabsorption, and when in excess, reduces the kidney’s ability to excrete water, leading to euvolemic hyponatremia (SIADH). Damage to neurons in the hypothalamic-hypophyseal tract allows the release of stored ADH [9]. Although damage to any part of this pathway can lead to excessive ADH secretion, the pituitary is the most common source following transsphenoidal resection [9]. Alternative hypotheses for the development of SIADH suggest that neurovascular compromise of the pituitary gland and hypothalamus, cerebral salt wasting, or low cortisol levels may contribute to postoperative hyponatremia [2,5,9,10].

Abnormal sodium levels after pituitary surgery have been described by a triphasic model, which begins with polyuria approximately 24-48 hours postoperatively, progresses to SIADH five to eight days postoperatively, and ends with a chronic polyuric phase. However, studies suggest that this triphasic response is rare [11]. More commonly, sodium disorders present as hypernatremia from AVP-D or hyponatremia from SIADH.

Our patient exhibited notable differences compared to previously reported cases of SIADH following transsphenoidal surgery. Most studies report hyponatremia onset between POD 4-7 [2,5,6,12], with few reporting onset on POD 1 [4,5]. Jahangiri et al. found that the incidence of hyponatremia was highest on POD 2 (46 of 1,045 patients), followed by POD 1 (33 of 1,045 patients) [4]. Kristof et al. reported a single patient out of 57 developing hyponatremia beginning on POD 1 [5]. These cases are atypical, as most studies report onset on POD 4-7 with a nadir occurring after day 7 [3,6]. Evidence suggests that the severity of hyponatremia correlates with the day of onset [10]. Barber et al. reported that mild hyponatremia (131-134 mmol/L) occurred earlier than moderate hyponatremia (125-130 mmol/L), although in their cohort, hyponatremia still began on POD 3-4 [10]. Our patient developed symptoms on POD 2. In one study, most patients developing hyponatremia on POD 1 were asymptomatic [4], with nausea being the most commonly reported symptom among symptomatic patients [4,10]. Patients with lower sodium nadirs are also more likely to exhibit symptoms [12].

Reliable predictors of postoperative hyponatremia have rarely been identified [3,10]. Some studies suggest that women are more likely to develop hyponatremia with a lower nadir compared to men [6,10]. Other studies have not identified significant differences based on age, gender, or tumor size [3]. Preoperative hypopituitarism has been reported as a predictor of postoperative hyponatremia; however, our patient had no evidence of hypopituitarism on preoperative evaluation [3,4,12].

Management of postoperative SIADH varies according to severity. Hyponatremia is often transient and, if mild or moderate, can be managed with fluid restriction or salt tablets [4,5]. Prior studies have suggested vasopressin receptor antagonists (e.g., tolvaptan) as a reasonable treatment option for severe hyponatremia [4].

## Conclusions

We describe a rare occurrence of early-onset hyponatremia, consistent with SIADH, following transsphenoidal pituitary resection, highlighting the importance of considering SIADH in both early and later postoperative periods. Early identification is critical due to the potential for serious adverse outcomes, including seizures and death. Treatment with fluid restriction, hypertonic saline, and salt tablets can effectively restore sodium levels in affected patients.
